# 3D visualization ablation planning system assisted microwave ablation for hepatocellular carcinoma (Diameter >3): a precise clinical application

**DOI:** 10.1186/s12885-020-6519-y

**Published:** 2020-01-20

**Authors:** Chao An, Xin Li, Min Zhang, Jian Yang, Zhigang Cheng, Xiaoling Yu, Zhiyu Han, Fangyi Liu, Linan Dong, Jie Yu, Ping Liang

**Affiliations:** 1 Department of Interventional Ultrasound, State Key Laboratory of Kidney Disease, The Chinese PLA General Hospital, No. 28, Fuxing Road, Beijing, 100853 People’s Republic of China; 2 Department of Ultrasound, General Hospital of Xinjiang Military Region, Urumqi, China; 3 Beijing Engineering Research Center of Mixed Reality and Advanced Display, School of Optics and Electronics, Beijing Institute of Technology, Beijing, 100081 China

**Keywords:** Hepatocellular carcinoma, Microwave ablation, 3D visualization ablation planning system, Local tumor progression, Overall survival

## Abstract

**Background:**

The aim of this retrospective study was to compare the feasibility and efficiency of ultrasound-guided percutaneous microwave ablation (US-PMWA) assisted by three-dimensional visualization ablation planning system (3DVAPS) and conventional 2D planning for hepatocellular carcinoma (HCC) (diameter > 3 cm).

**Methods:**

One hundred thirty patients with 223 HCC nodules (5.0 ± 1.5 cm in diameter, [3.0–10.0 cm]) who met the eligibility criteria divided into 3D and 2D planning group were reviewed from April 2015 to August 2018. Ablation parameters and oncological outcomes were compared, including overall survival (OS), recurrence-free survival (RFS), and local tumor progression (LTP). Multivariate analysis was performed on clinicopathological variables to identify the risk factors for OS and LTP.

**Results:**

The median follow-up period was 21 months (range 3–44). Insertion number (5.4 ± 1.2 VS. 4.5 ± 0.9, *P* = 0.034), ablation time (1249.2 ± 654.2 s VS. 1082.4 ± 584.7 s, *P* = 0.048), ablation energy (57,000 ± 11,892 J VS. 42,600 ± 10,271 J, *P* = 0.038) and success rate of first ablation (95.0% VS. 85.7%, *P* = 0.033) were higher in the 3D planning group compared with those in 2D planning group. There was no statistical difference in OS, and RFS between the two groups (*P* = 0.995, *P* = 0.845). LTP rate of 3D planning group was less than that of 2D planning group (16.5% VS 41.2%, *P* = 0.003). Multivariate analysis showed tumor maximal diameters (*P* < 0.001), tumor number (*P* = 0.003) and preoperative TACE (*P* < 0.001) were predictors for OS and sessions (*P* = 0.024), a-fetoprotein level (*P* = 0.004), and preoperative planning (*P* = 0.002) were predictors for LTP, respectively.

**Conclusions:**

3DVAPS improves precision of US guided ablation resulting in lower LTP and higher 5 mm-AM for patients with HCC lesions larger than 3 cm in diameter.

## Background

Microwave ablation (MWA) is an acceptable therapeutic efficiency option for hepatocellular carcinoma (HCC) with several advantages, such as produce larger ablation volume rapidly, less affected by heat-sink effect and less dependence on the electrical conductivities compared to radiofrequency ablation (RFA) [1–4]. However, high local tumor recurrence rates seriously restrict the long-term survival of HCC, especially for larger tumors or challenging locations ones [5–8]. A possible reason is the inability to determine the optimal permutation of ablations spheres and the exact location of antennae placement to completely destroy target tumors based on traditional two-dimensional (2D) preoperative treatment planning, which is mainly dependent on spatial awareness and highly subjective experience of radiologists. Therefore, how to achieve scientific, objective, quantizable and precise treatment planning is one of the key issues to US-PMWA for HCC.

Three-dimensional visualization ablation planning system (3DVAPS) is a commercially available soft by home grown, which is mainly based on segmentation and reconstruction of 2D image (CT or MRI). Multiple ablation spheres of fixed size covering target and spatial location relationship between tumor and surrounding vital organs can be displayed easily in the 3D visualization software. In addition, the application of 3D visualization technology allows radiologist to perform various manipulations in the 3D image, such as free locomotion, scaling and rotation to product a puncture plan by seeing more intuitively. 3DVAPS was originally applied to hepatectomy and began to be assistance in ablation in recent years [9–12], which has many sharp-cut characteristics including interactive manual simulating of the insertion number, display of virtual thermal field and calculation of the distance between the target and surrounding vital structures. In particular, the more precise calculation of residual liver ratio can effectively predict the liver function. These calculation results could improve ablation success and survival outcomes of patients with larger HCC underwent US-PMWA significantly. In this system, two functions related with assessment of ablative margin was as follows. Firstly, a 5 mm-AM covering target tumor was automatic generated in 3D display. Secondly, a 2D tumor map based on fusion between preoperative and postoperative 3D image, which improved assessment ability for complete ablation of target tumor. According to assessed result from tumor map, a second preoperative ablation planning was instituted.

The precise 3D mathematic model and AM of tumor map will be introduced in this study. Moreover, treatment parameters and oncologic outcomes of patients with HCC underwent US-PMWA were compared between 2D and 3D planning group. The purpose of this study is to evaluate the clinical application value of 3DVAPS in MWA for HCC (diameter > 3 cm).

## Methods

### Study design and patient data

Protocols involved in the current retrospective single-center study was devised based strictly on the principles of the Declaration of Helsinki and was approved by the Ethics Committee, allowing for informed consent to be waived. One hundred thirty consecutive patients (30 females, 100 males; average age 59.2 ± 10.3 years) with 223 HCCs who underwent US-PMWA from April 2015 to August 2018 were reviewed in electronic medical records. Before ablation, the preoperative planning was performed by three interventional radiologists (L.P., Z.G.C., and L.N.D who had 25, 25 and 5 years MWA experience). According to different types of plan, all eligible patients were divided into two groups including 3D group (i.e. preoperative planning with the aid of the self-developed 3DVAPS) and 2D group (i.e. preoperative planning according to traditional 2D image (CT/MRI)).

The diagnosis of HCC was performed according to the guidelines of the European Association for the Study of Liver and the American Association for the Study of Liver Disease [13]. Final diagnosis was confirmed based on histological evidence from needle biopsy specimens, which was operated before MWA in treatment of all patients. Patients were included in our study as follows: (a) had one HCC mass with maximum diameter larger than 3 cm, and less than 10 cm and number < 3, which were surrounded by a capsule at imaging or had smooth well circumscribed margins; (b) had sufficient contrast enhanced image (CT or MRI) data before and after MWA; (c) Child-Turcotte-Pugh (CTP) class A or B; (d) no evidence of extrahepatic metastasis or vascular invasion; (e) refuse to undergo hepatectomy or liver transplantation. The exclusion criteria were as follows: (a) had received other treatments (i.e., liver resection, liver transplantation, or iodine 125 seed implantation) beside transcatheter arterial chemoembolization (TACE) before treatment; (b) preoperative image data missing; (c) had serious medical comorbidities, including heart, lung and renal function dysfunction; (d) had severe coagulation disorders (i.e., prothrombin time > 25 s, prothrombin activity <40%, and platelet count <50 cells× 10^9^/L.); (e) active severe infection.

### 3D visualization preoperative ablation planning

**Fig. 1:**
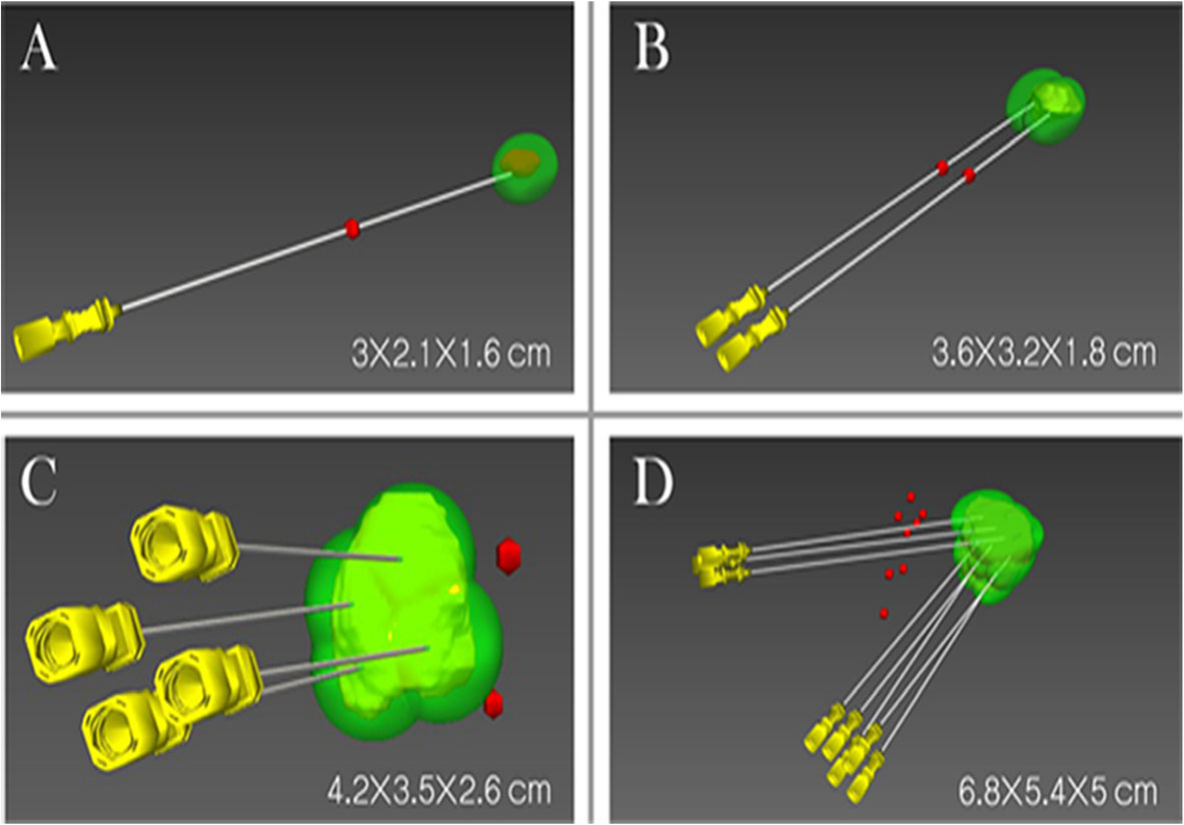
Different permutation and combination of microwave antenna according to shape and volume of the tumor in three-dimensional visualization ablation planning system. **a** A tumor size in 3.0 cmx2.1 cmx1.6 cm was completely covered by thermal field generated from one antenna. **b** A tumor size in 3.6cmx3.2cmx1.8 cm was completely covered by thermal field generated from simultaneously inserted by two antennas. **c** A tumor size in 4.2cmx3.5cmx2.6 cm was completely covered by thermal field generated from simultaneously inserted by four antennas. **d** A tumor size in 6.4 cmx5.8 cmx5cm was completely covered by thermal field generated from simultaneously inserted by eight antennas

A desktop computer (Lenovo) with an Intel Core i5 processor for an empirical study in our department was used to perform 3D visualization operative planning. A series of CT data (0.625-mm-thick slices) or MRI data (2.5-mm-thick slices) related with HCC before MWA were converted to DICOM format and then imported into the self- innovative 3DVAPS (made in Hokai company, Zhuhai, China). The details of 3DVAPS assisted MWA has been reported in previous studies (Additional file 1: Table S1). The graphical user interface displayed the real-time simulation ultrasound-guided MWA and the 3D visualization planning, as well as the planning path from the transverse, coronal and sagittal plane. The liver, target mass with a 5 mm-tumor-free margin, vessel and surrounding vital structures was segmented rapidly (within 2 min), which can stereo display in the 3D visualization. Target sphere volume and quantitative distance between the tumor and surrounding structures was calculated automatically. In the 3D model, the puncture path planning was interactive manual simulated accorded with the tumor size, location, the relationship between tumor and surrounding organs, and the common operating habit of interventional radiologists. The spherical thermal field was set up based manufacturer recommendation during preoperative planning [14]. According to the tumor size, different permutation and combination of ablation spheres were adjusted (Fig. 1), and the following principles must be complied: (a) for the distance between the tumor boundary and vital structures was larger than 5 mm, expanded ablation was applied; or else conformal ablation was applied. Expanded ablation is covering the entire tumor plus a predefined safety margin as much as possible. Conformal ablation is ablation zone covering the entire tumor only; (b) minimizing number of ablation spheres; and (c) minimizing inserting distance to target, avoiding puncture of critical structures along the path of insertion. The planning system was designed to iterate through these goals until a reasonable and feasible planning was achieved. Compared with 3D planning, 2D planning is simpler and subjective. Interventional radiologists visualize the needle path in the MRI or CT cross-sectional image depend on their experience.

### US-PMWA procedure

**Fig. 2:**
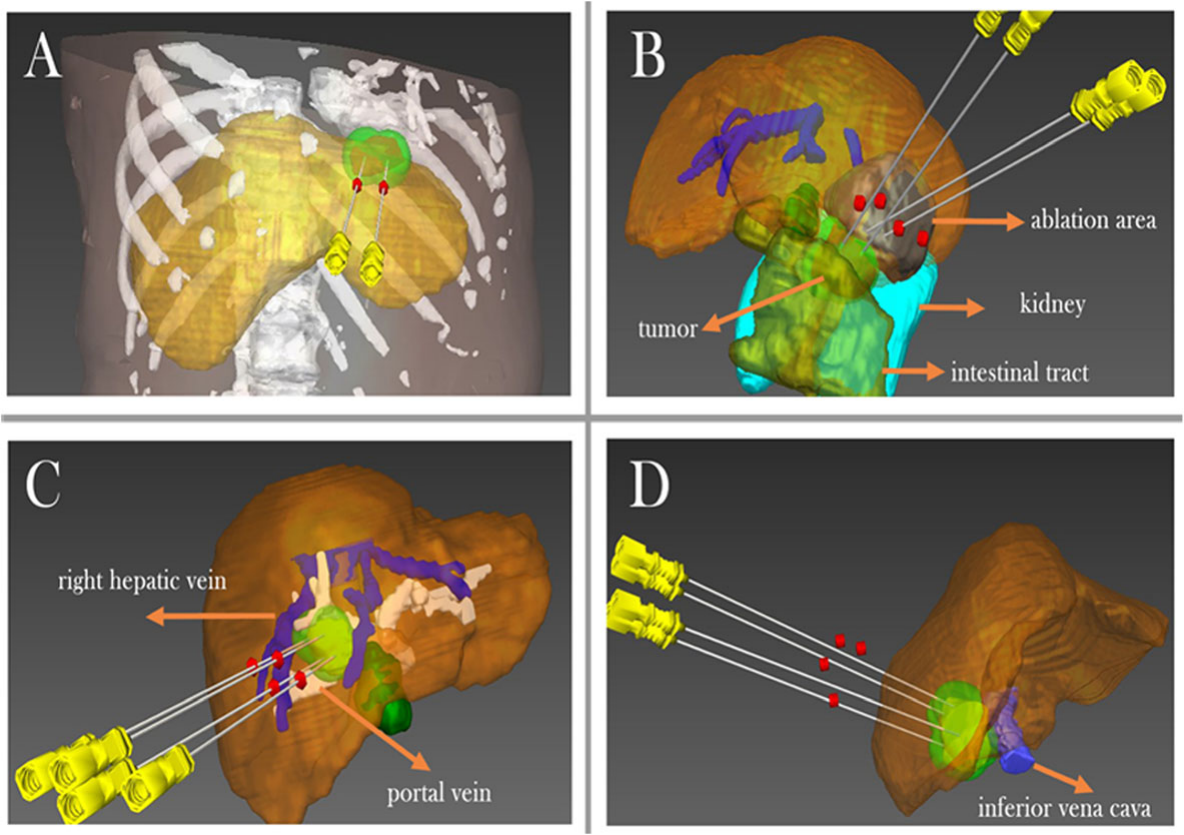
Tumor located in challenging locations was performed three-dimensional visualization preoperative planning before microwave ablation treatment. **a** A tumor abutting the diaphragm was covered by simulated thermal field used by two antennas. **b** A tumor abutting intestinal tract was covered by simulated thermal field used by four antennas. **c** A tumor abutting portal vein was covered by simulated thermal field used by four antennas. **d** A tumor abutting inferior vena cava was covered by simulated thermal field used by four antennas

**Fig. 3:**
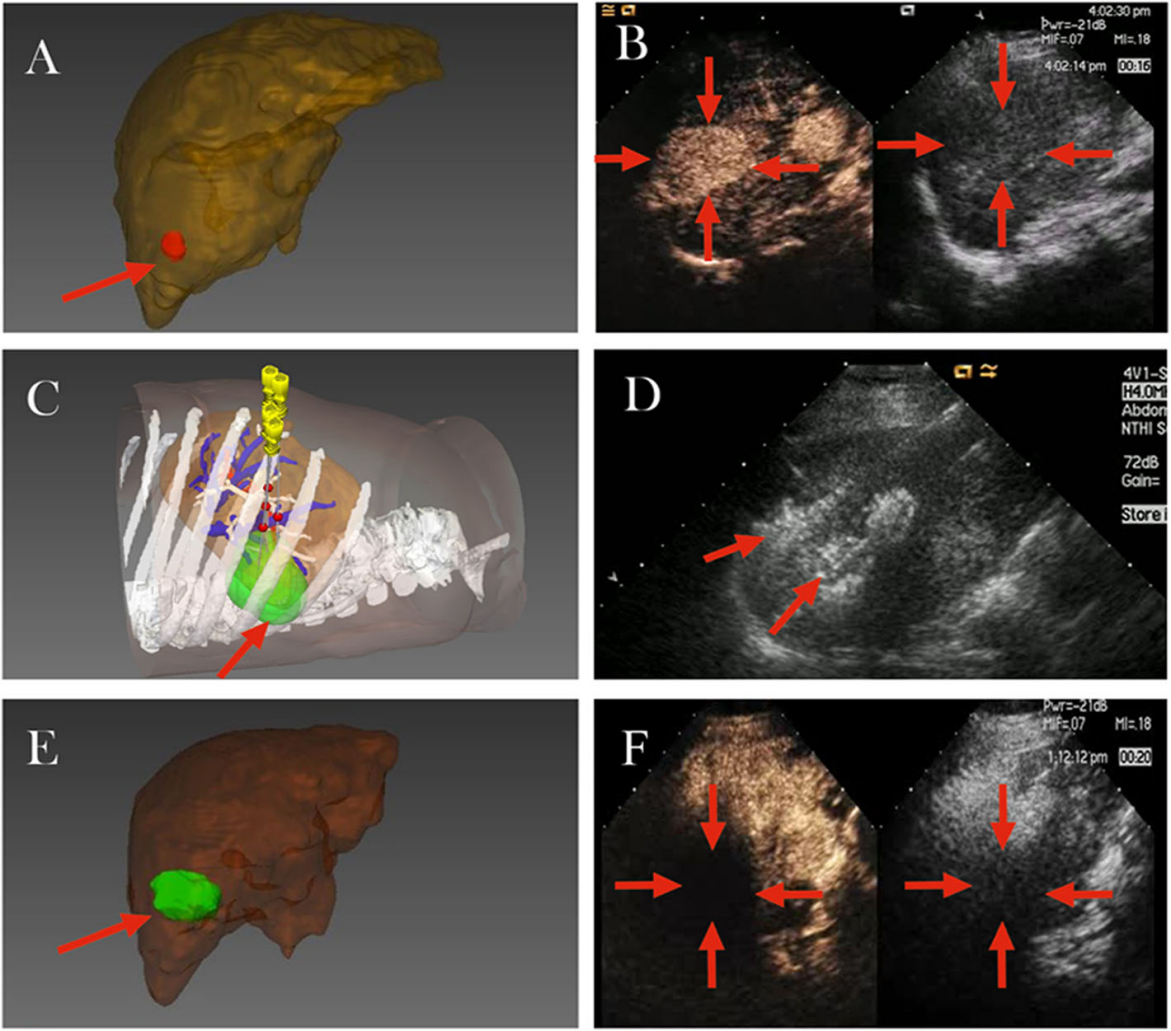
A patient (age older than 60 years) had a HCC tumor (2.4 cmx2.1cmx1.9 cm in diameter) located in S6 who underwent 3D visualization preoperative planning (**a-c**). The lesion (red) was segmented rapidly, which can stereo display in the 3D visualization before microwave ablation. The whole lesion was covered by suitable thermal field sphere generated from two antennas in 3D visualization ablation planning system. The ablation area (green) was segmented rapidly, which can stereo display in the 3D visualization after microwave ablation (**d-e**). A clear-boundary, regular-form tumor was showed in the CEUS image. According to 3D visualization preoperative planning, two antennas inserted in tumor under US-guided. Postoperative ablation area was showed in CEUS image

The US-PMWA operation was performed by three interventional radiologists (P. L., 20 years of experience, X.L.Y., 20 years of experience, and J.Y., 10 years of experience in MWA). The patients were under unconscious intravenous anaesthesia (propofol, 6-12 mg/kg/h; ketamine, 1-2 mg/kg) during the ablation in operating room. The tumors and tumor-feeding arteries were not well visualized with conventional US, a CEUS-guided ablation was performed. An automatic biopsy gun with an 18G cutting needle was used to carry out a US-guided biopsy immediately before ablation. Consequently, a 15G antennas was inserted directly into the tumor percutaneously. Multisite ablation was performed according to preoperative planning and MW power range was controlled between 50 W and 60 W. The antenna was then repeatedly inserted until the ablation zone achieved according to the planning. The masses were located in challenging locations main including tumors abutting the diaphragm, gastrointestinal tract, heart, major vessel (i.e. portal vein, hepatic vein and inferior vena cava), which need to more precise puncture based on 3D visualization mathematical model (Fig. 2). Contrast-enhanced ultrasound (CEUS) with Sono Vue (Bracco, Milan, Italy) was performed to target the tumor accurately preoperatively and assess the postoperative ablation effect immediately after ablation (Fig. 3).

### Construction of tumor map

3D visualization image registration used with same image data before and after MWA. Segmented procedure of tumor used in the arterial phase and ablation areas in the delay phase. The registration process has been documented in our previous study [15]. A tumor map with traffic light color scheme was used to emphasize ablative effect (Additional file 2: Figure S1). Red was defined as residual tumor and ablation field uncovering target; yellow was defined as ablation field covering target but fail to achieve 5 mm-AM and green was defined as ablation field covering target with achieving 5 mm-AM.

### Determination of survival, local tumor progression, technique effective and complications

Death and recurrence were taken as the study endpoints. Overall survival (OS) represented the duration between the date of first MWA treatment session to the last follow-up date (either survival or lost to follow-up) or upon patient death. Recurrence-free survival (RFS) was taken as the duration between the date of first MWA treatment session to the last follow-up date (either survival or lost to follow-up) or date of tumor recurrence. LTP was defined as the appearance of irregular nodular, scattered, or eccentric pattern of peripheral enhancement around the ablation zone after MWA. Technique effectiveness was defined as the absence of enhancement of any areas of the mass at a follow-up contrast enhanced image examination performed 1 month after MWA. Complications were classified according to the Society of Interventional Radiology Classification system for complications by outcome [16].

### Follow-up

To assess treatment efficacy, contrast-enhanced multiphase images (or computed tomography [CT] and magnetic resonance imaging [MRI]) were performed 1 week after the last course of a defined ablation protocol. In patients who were deemed to have undergone sufficient ablation, either an MRI or contrast-enhanced CT along with serum tumor markers were repeated at 1 and 3 months post-MWA, as well as at intervals between 3 and 6 months. Lesions suspicious for metastasis were further investigated with a thoracic CT, bone scan or PET-CT. After the study duration, patients were free to determine their subsequent treatment options, with radiologist-guided advice. Those who developed recurrence were treated via surgical resection, MWA, radiofrequency ablation (RFA), transcatheter arterial chemoembolization (TACE), or systemic chemotherapy, based on patients’ overall health status, lesion location and liver function.

### Statistical analysis

Continuous variables were analyzed with the Mann-Whitney U test while the Pearson χ2 analysis or Fisher exact tests were used to analyze categorical variables. OS, RFS and LTP rates were then derived using the Kaplan-Meier method with log-rank test. Univariate and multivariate analyses of independent risk factors were assessed based on the forward stepwise Cox regression model. A nomogram was constructed using the Cox-model derived β coefficients in order to delineate the association between clinical variables and oncological outcomes Internal validation with 1000 sets of bootstrap samples was performed to evaluate the use of the nomogram for assessment of OS and LTP of patients who underwent MWA. The SPSS 22.0 (SPSS, Chicago, IL) program and the RMS package of the R software version 3.5.1 (http://www.r-project.org/) were used for all statistical analysis. A two-sided *P* value <0.05 was taken to represent statistical significance.

## Results

### Baseline characteristics and treatments parameters

**Fig. 4:**
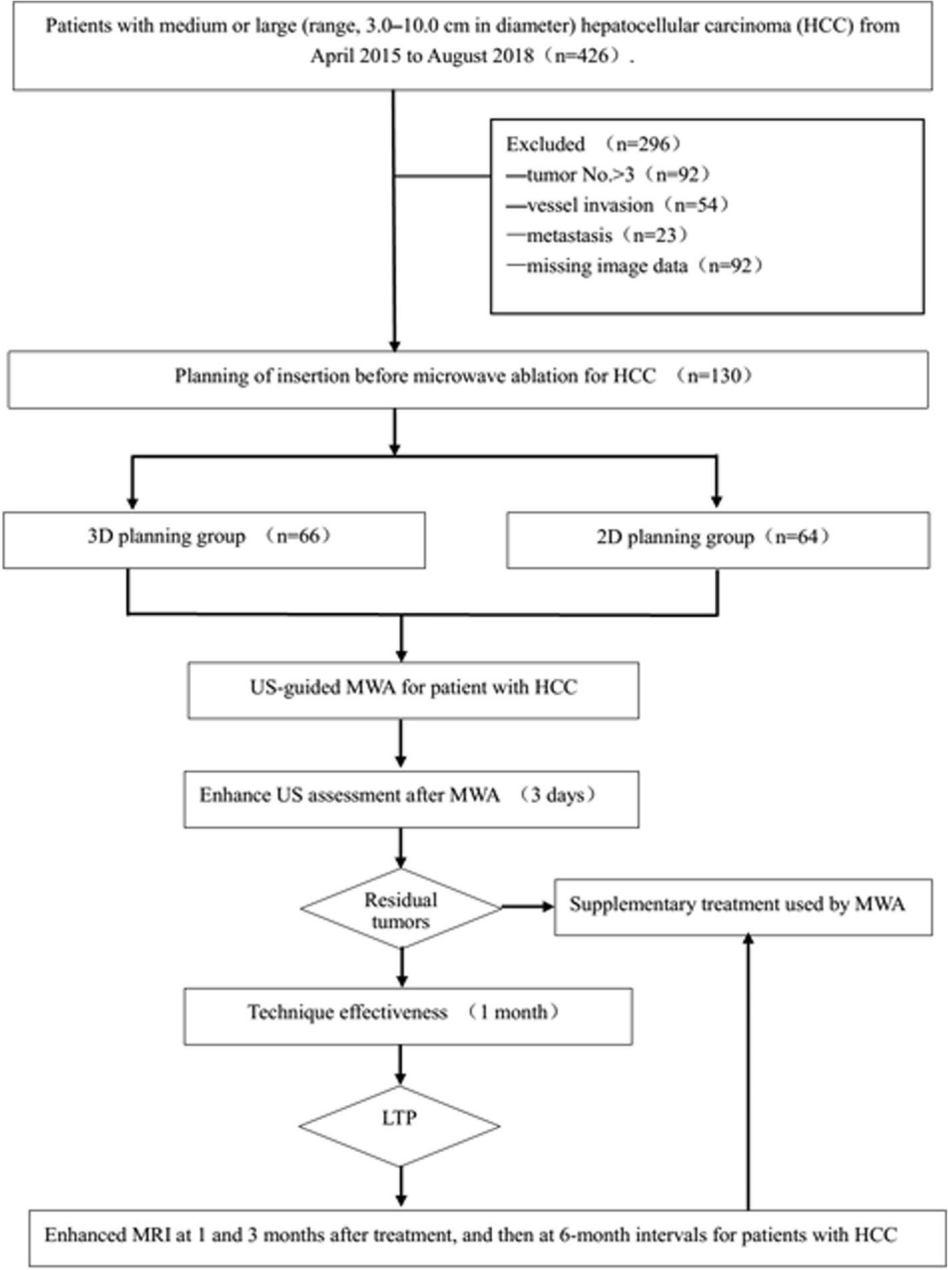
Flow diagram shows study patient accrual process

A total of 426 patients with HCC (3-10 cm in diameter) underwent US-PMWA during the study period. As evaluated by the flow chat in Fig. 4, a total of 296 patients were excluded because they could not met the inclusion criteria. As a result, 66 patients with 121 HCCs (12 females, 54 males; average age 60.6 ± 11.7 years) in 3D planning group and 64 patients with 102 HCCs (16 females, 48 males; average age 58.6 ± 10.0 years) in 2D planning group were recruited. Characteristics of the patients and tumors are presented in Table 1. Significant statistical difference was detected in tumor location (in left or right or both liver lobe) between two groups (*P* = 0.033). The tumors in challenging locations includes 7 abutting major vessel, 12 abutting diaphragm and 14 abutting gastrointestinal tract in 3D planning group, which compared with those of 10 abutting major vessel, 17 abutting diaphragm and 12 abutting gastrointestinal tract in 2D planning group (*P* = 0.454). So hydrodissection and thermal monitoring techniques were applied in 72 patients with 100% one-time success rate. Image data imported into 3DVAPS were CT and MRI data, including 24 CT data and 42 MRI data in 3D planning group and 30 CT data and 34 MRI data in 2D planning group. Mean tumor volume (65.6 ± 21.2 ml VS 62.8 ± 28.3 ml, *P* = 0.295), mean ablation area volume (145.7 ± 89.1 ml VS 157.2 ± 95.5 ml, *P* = 0.527), residual liver ratio (89.5 ± 21.2% VS 87.3 ± 25.7%, *P* = 0.772) were compared between 3D planning and 2D planning groups. All patients underwent ablation accordingly to 2D or 3D preoperative planning successfully. The ablation parameters were presented in Table 1. One-hundred and thirty patients with 223 HCCs received a total of 278 sessions of ablation. Insertion number (5.4 ± 1.2 VS. 4.5 ± 0.9, *P* = 0.034), ablation time (1249.2 ± 654.2 s VS. 1082.4 ± 584.7 s, *P* = 0.048), ablation energy (57,000 ± 11,892 J VS. 42,600 ± 10,271 J, *P* = 0.038) and success rate of first session (SRFS) rate (95.0 ± 11.2% VS. 85.7 ± 9.4%, *P* = 0.033) in 3D planning group were significantly higher than that in 2D planning group.

**Table 1:** Characteristics of Patients, Tumors and Ablation Parameters

Characteristic	3D planning group (*n* = 66)	2D planning group (*n* = 64)	*P* Value
Age (y)			0.296
Mean ± SD	60.6 ± 11.7	58.6 ± 10.0	
Range	36–81	34–76	
Sex			0.898
Male	52 (78.8)	48 (75)	
Female	14 (21.2)	16 (25)	
Comorbid disease			0.835
No	28 (42.4)	26 (40.6)	
Yes	38 (57.6)	38 (59.4)	
Pathological differentiation			0.597
Well/moderately	32 (48.5)	34 (53.1)	
Poorly	34 (51.5)	30 (46.9)	
Etiology			0.381
No	5 (7.6)	2 (3.1)	
HBV	59 (89.4)	58 (90.7)	
HCV	2 (3.0)	4 (6.2)	
Cirrhosis			0.124
Yes	55 (83.3)	59 (92.2)	
No	11 (16.7)	5 (7.8)	
Maximal tumor diameter (cm)			
Mean ± SD	5.0 ± 1.5	5.0 ± 1.6	0.721
Range	3.0–8.7	3.0–10.0	
3.1–4.9	47 (71.2)	37 (59.1)	0.147
5.0–6.9	11 (16.7)	20 (30.3)	
7.0–10.0	8 (12.1)	7 (10.6)	
Tumor no.			0.987
1	36 (54.5)	35 (54.7)	
2–3	30 (45.5)	29 (45.3)	
Tumor volume (ml)			0.295
Mean ± SD	65.6 ± 21.2	62.8 ± 28.3	
Range	6.32–171.12	7.73–182.6	
<62.5	51 (75.8)	48 (71.9)	
≥ 62.5	15 (24.2)	16 (28.1)	
Approximated sphericity			0.491
Yes	12 (18.2)	10 (15.7)	
No	54 (81.8)	54 (84.3)	
Ablation volume (ml)			0.772
Mean ± SD	145.7 ± 89.1	157.2 ± 95.5	
Range	26.3–296.6	24.5–312.8	
Residual liver ratio (%)			0.527
Median	89.5 ± 21.2	87.3 ± 25.7	
Range	63.9–98.0	72.1–96.8	
Location			0.030*
Left liver lobe	7 (10.6)	18 (28.3)	
Right liver lobe	56 (84.8)	45 (68.2)	
Left + Right liver lobe	3 (4.6)	1 (1.5)	
Adjacent to organ			0.454
No	33 (50)	25 (39.1)	
Major vessels	7 (10.6)	10 (15.6)	
Diaphragm	12 (18.2)	17 (25.8)	
Gastrointestinal tract	1(4 21.2)	12(18.2)	
CTP grade			0.208
A	61 (92.4)	63 (98.4)	
B	5 (7.6)	1 (1.6)	
α-fetoprotein level (ng/ml)			0.382
> 400	30 (45.5)	34 (53.1)	
≤ 400	36 (54.5)	30 (46.9)	
Preoperative image type			0.429
CT	24 (36.4)	30 (46.9)	
MRI	42 (63.6)	34 (53.1)	
Chemoradiation			0.812
Yes	59 (89.4)	58 (90.6)	
No	7 (10.6)	6 (9.4)	
Preoperative TACE			0.117
Yes	11 (16.7)	18 (28.1)	
No	55 (83.3)	46 (71.9)	
Complications			0.712
Yes	5 (7.6)	6 (9.4)	
No	61 (92.4)	58 (90.6)	
Follow-up (months)			0.287
Median	17.7	24.2	
Range	4.3–43.7	2.9–42.2	
Postoperative Metastasis			0.447
Yes	9 (13.6)	6 (9.4)	
No	57 (86.4)	58 (90.6)	
Antenna number			0.391
2	112 (84.8)	98 (81.3)	
> 2	9 (15.2)	4 (18.7)	
Insertion number			0.034*
Mean ± SD	5.4 ± 1.2	4.5 ± 0.9	
Range	2–12	2–11	
Ablation time (s)			0.048*
Mean ± SD	1249.2 ± 654.2	1082.4 ± 584.7	
Range	380–3360	280–2290	
Ablation power (W)			
Mean ± SD	60 ± 5	60 ± 7	0.725
Range	40–65	40–65	
Ablation energy (J)			0.038*
Mean ± SD	57,000 ± 11,892	42,600 ± 10,271	
Range	12,000–220,800	12,000–20,600	
Ablation frequency (Hz)			0.482
915	6 (6.1)	3 (4.7)	
2450	115 (93.9)	99 (95.3)	
Sessions			0.033*
1	115 (95.0)	87 (85.3)	
> 1	6 (5.0)	15 (14.7)	

Note.-Except where indicated, data are numbers of patients. Data in parentheses are percentages and were calculated by using the total number of patients in each group as the denominator. *SD* Standard deviation, *TACE* Transarterial chemoembolization, *CT* Computed tomography, *MRI* Magnetic resonance imaging

### Oncological outcomes after US-PMWA

**Fig. 5:**
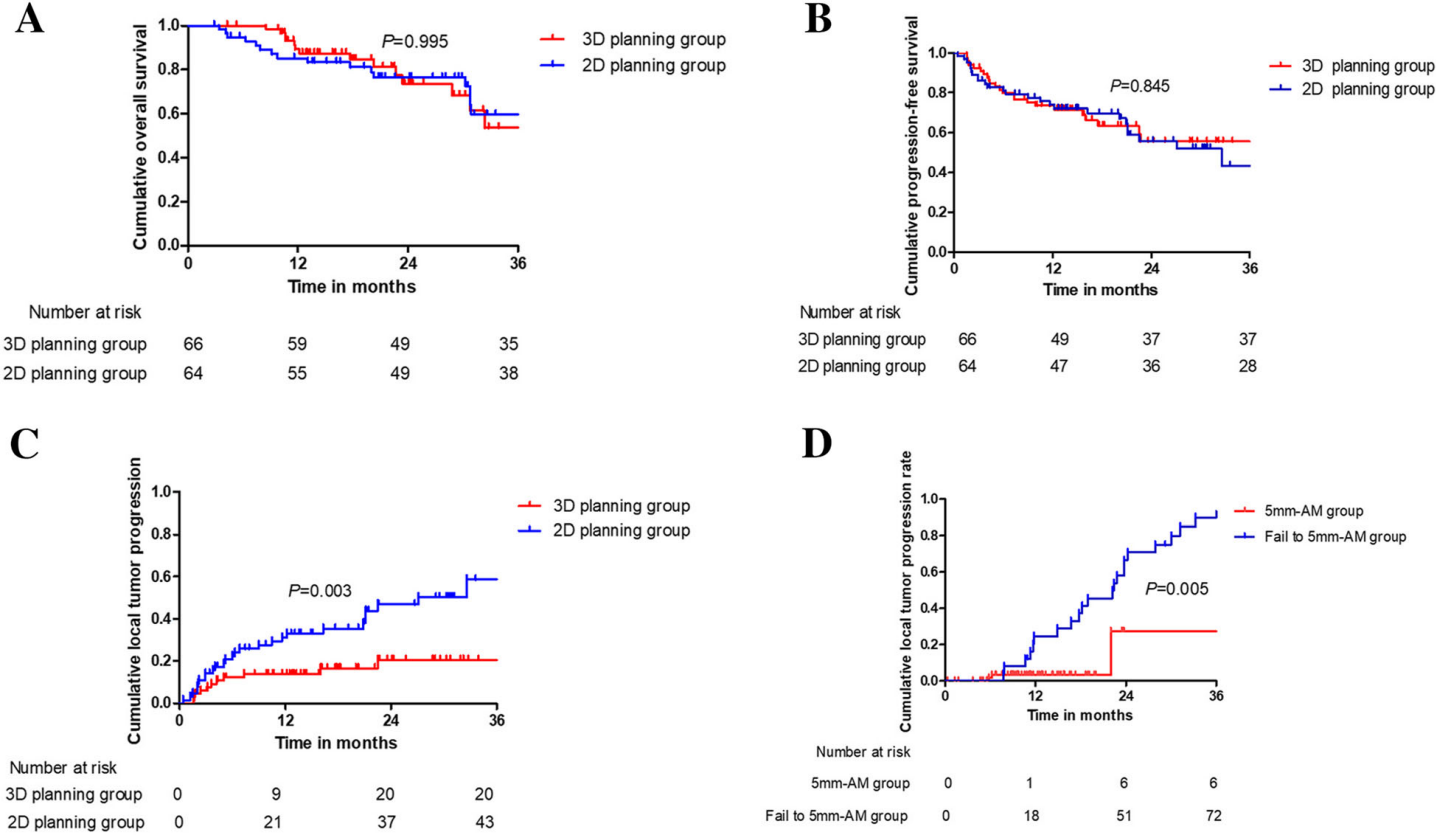
**a** The 1-, 2-, and 3-year OS rates of 3D group and 2D group were 89.3, 73.5, 53.7 and 85.2%, 76.4, 59.6%, respectively, showing no significant statistical difference (*P* = 0.995). **b** The 1-, 2-, and 3-year RFS rates of 3D group and 2D group were 73.5, 55.6, 55.6 and 73.9%, 55.9, 43.5%, respectively, showing no significant statistical difference (*P* = 0.845). **c** The 1-, 2-, and 3-year LTP rates of 3D group and 2D group were 13.8, 20.6, and 20.6% and 31.2, 46.8, 58.6%, respectively, showing significant statistical difference (*P* = 0.003). **d** According to tumor map, the 1-, 2-, and 3-year LTP rate of achieved to 5 mm-AM group and failed achieving to 5 mm-AM group were 3.2, 22.4, 27.4 and 24.6%, 66.5, 89.9%, respectively, showing significant statistical difference (*P* = 0.005)

The median follow-up period was 21 months (range 3–44 months). The median survival periods were 22 months (6–44 months) in 3D planning group and 20 months (8–43 months) in 2D planning group (*P* = 0.995). Oncologic outcomes of follow-up period after US-PMWA between 3D planning group and 2D planning group are presented in Table 2. On the basis of follow-up image, no significant statistical difference was detected in technique effectiveness rate between 3D planning group and 2D planning group (98.3% VS 97.1%, *P* = 0.492). The 1-, 2-, and 3-year OS rates of 3D planning group and 2D planning group were 89.3, 73.5, 53.7 and 85.2%, 76.4, 59.6%, respectively (Fig. 5a), showing no significant statistical difference (*P* = 0.995). No significant statistical difference was detected in the 1-, 2-, and 3-year RFS rate of 3D planning group and 2D planning group with 73.5, 55.6, 55.6 and 73.9%, 55.9, 43.5%, respectively (*P* = 0.845) (Fig. 5b). While the 1-, 2-, and 3-year LTP of 3D planning group and 2D planning group were 13.8, 20.6, and 20.6% and 31.2, 46.8, 58.6%, respectively (Fig. 5c), showing significant statistical difference (*P* = 0.003).

**Table 2:** Outcomes of Follow-up after MWA Between 3D Planning Group and 2D Planning Group

Parameters	3D planning group (*n* = 121)	2D planning group (*n* = 102)	*P* Value
Technique effectiveness			0.492
Yes	119 (98.3)	99 (97.1)	.
No	2 (1.7)	3 (2.9)	
Success of first session			0.033*
Yes	115 (95)	87 (85.3)	
No	6 (5)	15 (14.7)	
Local tumor progression			0.003*
Yes	20 (16.5)	42 (41.2)	
No	101 (83.5)	60 (58.8)	
Recurrence			0.845
Yes	43 (35.5)	50 (49)	
No	78 (64.5)	52 (51)	
Death			0.995
Yes	31 (46.4)	28 (40.4)	
No	35 (53.7)	3(6 59.6)	
Major complications			0.762
Yes	5 (9.1)	6 (9.4)	
No	61 (90.9)	58 (90.6)	

# Data in parentheses are percentages

* P less than 0.05 was considered as significant difference

### Univariate and multivariate analysis

Univariate and multivariate logistic regression analysis were performed to identify predictors influencing the long-term outcomes of patients with medium or large HCC who underwent US-PMWA. The univariate analysis showed statistically significant differences in terms of OS rates, depending on the age (χ^2^ = 4.153; *P* = 0.042), maximal tumor diameter (χ^2^ = 16.466; *P* < 0.001), tumor volume (χ^2^ = 11.275; *P* = 0.001), CTP grade (χ^2^ = 6.321; *P* = 0.012), and preoperative TACE (χ^2^ = 26.433; *P* < 0.001). The multivariate analysis showed that the factors that significantly affected the OS rate were maximal tumor diameter (HR = 7.782; *P* < 0.001), tumor number (HR = 0.343; *P* = 0.003), and preoperative TACE (HR = 5.968; *P* = 0.002) (Table 3). The univariate analysis showed statistically significant differences in terms of LTP rates, depending on the tumor volume (χ^2^ = 11.275; *P* = 0.001), tumor number (χ^2^ = 4.230; *P* = 0.040), α-fetoprotein level (χ^2^ = 7.439, *P* = 0.006), and preoperative planning (χ^2^ = 8.604; *P* = 0.003). The multivariate analysis showed that the factors that significantly affected the LTP rate were sessions (HR = 0.287; *P* = 0.027), α-fetoprotein level (HR = 2.644; *P* = 0.004) and preoperative planning (HR = 8.604; *P* = 0.003), (Table 4).

**Table 3:** Factors Associated with Overall Survival by Univariable and Multivariable Analysis

Factors	No.	Univariable Analysis		Multivariable Analysis	
		χ^2^	*P* Value	Hazard Radio	*P* Value
Age (years)		4.153	0.042*	…	…
< 65	68				
≥ 65	62				
Gender		0.040	0.842	…	…
Male	100				
Female	30				
Differentiation		1.069	0.820	…	…
Well/moderately	54				
Poorly	76				
Comorbidities		0.661	0.416	…	…
Yes	76				
No	54				
Etiology		0.472	0.065	…	…
No	13				
HBV	106				
HCV	11				
Cirrhosis		1.293	0.728	…	…
Yes	114				
No	16				
Tumor Maximal diameter (cm)		16.466	< 0.001*	7.782 (2.942–19.264)	< 0.001*
3–5	84				
> 5	46				
Tumor volume (ml)		11.275	0.001*	…	…
≤ 62.5	93				
> 62.5	37				
Tumor number		0.249	0.618	0.343(0.143–0.836)	0.003*
Single	71				
Multiple	59				
Adjacent major organ		2.801	0.278	…	…
Large vessels	17				
Diaphragm	29				
Gastrointestinal tract	26				
No	58				
Location		1.991	0. 370	…	…
Left liver lobe	25				
Right liver lobe	101				
Left + Right liver lobe	4				
CTP grade		6.312	0.012*	…	…
A	124				
B	6				
α-fetoprotein level (ng/ml)		3.176	0.075	…	…
> 400	64				
≤ 400	66				
Preoperative TACE		26.433	<0 .001*	8.882 (3.698–21.334)	<0 .001*
Yes	29				
No	101				
Sessions		0.834	0.361	…	…
1	110				
> 1	20				
Preoperative planning		0.520	0.821	…	…
3D	66				
2D	64				

Note. Date in parentheses are 95% confidence intervals. *TACE* Transarterial chemoembolization, *CTP* Child-Turcotte-Pugh, *3D* Three dimensional planning, *2D* Two dimensional planning. * P less than 0.05 was considered as significant difference

**Table 4:** Factors Associated with Local Tumor Progression by Univariable and Multivariable Analysis

Factors	No.	Univariable Analysis		Multivariable Analysis	
		χ^2^	*P* Value	Hazard Radio	*P* Value
Age (years)		0.106	0.745	…	…
< 65	68				
≥ 65	62				
Gender		1.124	0.289	…	…
Male	100				
Female	30				
Differentiation		2.229	0.337	…	…
Well/moderately	54				
Poorly	76				
Comorbidities		0.759	0.384	…	…
Yes	76				
No	54				
Etiology		0.455	0.797	…	…
No	13				
HBV	106				
HCV	11				
Cirrhosis		3.022	0.082	…	…
Yes	114				
No	16				
Maximal tumor diameter (cm)		3.162	0.075	…	…
3–5	84				
> 5	46				
Tumor volume (ml)		11.275	0.001*	…	…
≤ 62.5	93				
> 62.5	37				
Tumor number		4.230	0.040*	…	…
Single	71				
Multiple	59				
Adjacent major organ		0.904	0.636	…	…
Large vessels	17				
Diaphragm	29				
Gastrointestinal tract	26				
ND	58				
Location		5.962	0. 062	…	…
Left liver lobe	25				
Right liver lobe	101				
Left + Right liver lobe	4				
CTP grade		0.167	0.683	…	…
A	124				
B	6				
α-fetoprotein level (ng/ml)		7.439	0.006*	2.644 (1.354–5.160)	0.004*
> 400	64		.		
≤ 400	66				
Preoperative TACE		0.805	0.370	…	…
Yes	29				
No	101				
Sessions		1.897	0.184	0.287 (0.097–6.645)	0.024*
1	110				
> 1	20				
Preoperative planning		8.604	0.003*	3.217 (1.557–3.342)	0.002*
3D	66				
2D	64				

Note. Date in parentheses are 95% confidence intervals. *TACE* Transarterial chemoembolization, *CTP* Child-Turcotte-Pugh, *3D* Three dimensional, *2D* Two dimensional, *ND* No data. P less than 0.05 was considered as significant difference

### Postoperative evaluation by tumor map

Due to preoperative and postoperative contrast enhanced image data must be matched, the ablation effect of 72 treatment-naïve patients with 107 HCCs could be evaluated by tumor map according to inclusion criteria. Among them, 6 lesions with residual tumors with red color in tumor map were detected, 76 were evaluated having achieved to 5 mm-AM with green color and 25 were assessed failed in achieving to 5 mm-AM with yellow color. These target tumors were divided into two groups (i.e. achieved to 5 mm-AM group and failed achieving to 5 mm-AM group). The 1-, 2-, and 3-year LTP rate of the group achieved to 5 mm-AM and the group failed to achieve to 5 mm-AM were 3.2, 22.4, 27.4 and 24.6%, 66.5, 89.9%, respectively, showing significant statistical difference (*P* = 0.005) (Fig. 5d).

### Construction and validation of the nomogram

**Fig. 6:**
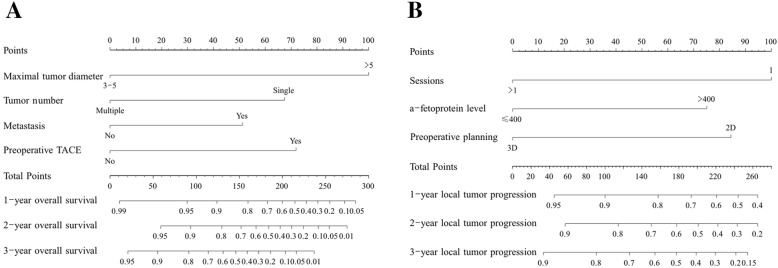
Nomogram shows assessment of 1-, 2- and 3-years OS and LTP of patients with HCC who underwent US-PMWA (**a**, **b**)

The nomogram was constructed by using β-coefficients to determine the proportional prognostic effect of the four independent risk factors to assess their association with OS and LTP in the multivariate analysis. Each enrolled patient received one individualized grade, which was the sum of the points from these prognostic variables. The projections from total points (range, 0–300) shown on the scales in Fig. 6a indicated the estimated probability of OS at 1-, 2- and 3-years. The concordance index for the model for assessment of OS after MWA was 0.811 and with 1000 cycles of bootstrapping (95% CI: 0.728–0.894). The projections from total points (range, 0–260) shown on the scales in Fig. 6b indicated the estimated probability of LTP at 1-, 2- and 3-years. The concordance index for the model for assessment of LTP after MWA was 0.693 and with 1000 cycles of bootstrapping (95% CI: 0.608–0.778).

## Discussion

In the past few years, US-PMWA has been developed as a promising minimal invasive alternative for treatment of HCC in early stage, which achieved satisfactory oncologic outcome [17–19]. However, to treat larger tumors, multiple antennas array is often performed during the procedure, which potentially resulted in an insufficient ablation [20, 21]. In order to improve low spatial resolution in 2D image planning, the 3D visualization of target tumor and potential risk structures such as major vessels or important organs gives essential support in 3DVAPS. In previous studies, 3D visualization technology had applied to treatment for liver cancer. For example, in hepatectomy, Hu M et al. reported that right posterior lobe allied with part of V and VIII sectionectomy assisted by 3D visualization technology, which could provide better preoperative strategy for surgeon [22]. Zhang J et al. reported that 3D visualization preoperative planning assisted surgical resection for progressive hilar cholangiocarcinoma, which improve the success rate of operation [23]. In addition, 3D visualization technology is also favored in image-guided interventional therapy. Liu F et al. have reported the clinical application value of a 3D visualisation preoperative treatment planning system in microwave ablation for liver cancer [12]. Li X et al. reported US-PMWA assisted by 3D visualisation preoperative treatment planning system and percutaneous transhepatic cholangial drainage with intraductal chilled saline perfusion for larger hepatic hilum HCC, which improved the local tumor control rate [24]. Given the characteristics of US-PMWA, including poor image quality affect from air-containing organ and subjectivity of intraoperative puncture, it is essential to set up a scientific, reasonable, quantizable and precision needle position protocol with 3DVAPS.

There were several major findings in our study by comparing the results between 2D planning with 3D planning groups Firstly, higher success rate of first session was achieved in the 3D planning group. Insertion number, ablation time and ablation energy were higher in 3D planning group when comparing with those in 2D planning group. These results are consistent with those of previous studies [10–12], which due to more precise and reasonable needle position protocol with 3DVAPS. Secondly, lower LTP rate in the 3D planning group than that in 2D planning group. Whereas OS and RFS rate was compared between two groups, which suggests that target tumor plus 5 mm-AM was easier to discern and covered by virtual thermal field in 3DVAPS. Thirdly, a tumor map exhibited reliable discriminative ability for assessment the ablation effect, which suggests that this simple and quantizable assessment could provide an important reference for the re-treatment of patients with larger HCC and assessment of LTP. Finally, we constructed two nomograms that can be used for individualized assessment of OS and LTP in patients with medium or large HCC who underwent US-PMWA.

Medium and large HCC with US-PMWA both were reported in previous studies. Among them, Giorgio A et al. reported that 3-year OS rate of patients with intermediate HCC underwent MWA was 60% and success rate of first session was 89% [25]. Zhang NN.et al. reported that high-powered microwave ablation for larger hepatocellular carcinoma (3-8 cm), which had 2-years OS was 86.67% and success rate of first session was 82.61% [26]. In our study, 2-years OS was 73.5% and success rate of first session was 95.0% in 3D group. Our cohort might have had a lower 2-year OS rate. However, when we considered the differences in patient number, abutting major vessel or organ, viral etiology, liver functional reserve and preoperative TACE in this study, we think it was not appropriate to compare our study results directly with those of previous studies. Moreover, higher RFS rate and lower LTP rate in 3D group suggested that precision and feasibility of 3DVAPS assist to US-PMWA for larger HCC. As 5 mm-AM was an important factor for controlling LTP, a comparison was performed according the tumor map in 3D image. The results indicated that the LTP in the group achieving 5 mm-AM was lower than that in the group fail to achieving 5 mm-AM, especially for larger HCC and tumor at a risk/challenging location [27]. In the multivariate analysis, maximal tumor diameter (diameter > 5 cm), tumor number (*n* > 1), and without preoperative TACE predicated poor OS for HCC. The results were in accordance with the previous report [28, 29]. For LTP, the multivariate analysis showed that more sessions, lower α-fetoprotein level and 3D planning were associated with better LTP controlling, which indicated that precision of treatment was vital in HCC.

In this study, we constructed a nomogram related to individualized risk estimations for OS of patients who underwent MWA for medium or large HCC. This nomogram demonstrated a high discriminatory ability with an ideal concordance index of 0.811 and was capable of generating individualized risk estimations for OS after MWA. The straightforward graphical tool consisted of ordinary clinical variables, including maximal tumor diameter, tumor number and preoperative TACE. The clinical variables included tumor factors and therapeutic alliance. Several reports had pointed out TACE combined with MWA was safe and effective in the treatment of large HCC lesions [30–32]. Furthermore, the convenience of using nomogram derived from pretreatment clinical variables to assess prognosis for individual patients with HCC before initiation of treatment may improve patient-physician communication, decision making and selection of patients for prospective clinical trials. In addition, another nomogram related to individualized risk estimations for LTP was constructed, including sessions, α-fetoprotein level and preoperative planning. α-fetoprotein level was a tumor marker that represents the tumor status, however, sessions and preoperative planning associated with US-PMWA treatment were more important for LTP, the reason for those was as following: firstly, more ablation session was feasible and effective treatment for larger HCC, and one session fail to complete ablation or achieve 5 mm-safety boundary easily, due to larger ablation area may result in poor liver functional reserve. Secondly, 3D preoperative planning had improved the accuracy of the positioning and may have strengthened radiologist’ confidence in ablation therapy.

There were several limitations to our study. Firstly, this was a single-center retrospective study, while patients with medium or large HCC (D > 3 cm) were included. So, a robust nomogram should be validated externally in different patient cohorts. Secondly, only 22.3% (29/130) patients accept TACE before US-PMWA, this combination treatment may improve OS and LTP rate than MWA treatment only. Thirdly, the treatment planning system does not account for cooling effect due to large vessel heat sinks. Finally, tumor map was showed in this study, but was not apply in assessment the efficiency of US-PMWA for medium or large HCC assisted. So further investigation was needed.

## Conclusions

Compared with the 2D planning group, 3D planning group had a higher success rate of first ablation and less sessions, which has a relatively high clinical application value for HCC (diameter > 3 cm). The 5 mm-AM was showed by tumor map in 3D image provided more information for controlling LTP. Therefore, the 3DVAPS provides a scientific, objective, quantizable and precise strategy, which could expand the indications of MWA in treatment of HCC.

## Supplementary information

**Additional file 1: Table S1.** Published literature related to 3DVAPS assisted MWA in our team.

**Additional file 2: Figure S1.** A picture of the procedure used for 3D visualisation image fusion and tumor map generation.

## Data Availability

The datasets generated and/or analyzed during the current study will be available on reasonable request by contacting Dr. Chao An (anchao-1983@163.com). And all data has been uploaded in this system as a supplementary information file.

## Abbreviations

2D: Two-dimensional
3DVAPS: Three-dimensional visualization ablation planning system
AM: Ablative margin
CEUS: Contrast-enhanced ultrasound
HCC: Hepatocellular carcinoma
LTP: Local tumor progression
OS: Overall survival
RFA: Radiofrequency ablation
RFS: Recurrence-free survival
TACE: Transcatheter arterial chemoembolization
US-PMWA: Ultrasound-guided percutanous microwave ablation
