# Supplementary material for: 3D visualization ablation planning system assisted microwave ablation for hepatocellular carcinoma (Diameter >3): a precise clinical application
Source: BMC Cancer. 2020 Jan 20;20:44. doi: 10.1186/s12885-020-6519-y (PMC6972027; doi:10.1186/s12885-020-6519-y)
Supplement: Supplementary file 1 — Additional file 1: Table S1. Published literature related to 3DVAPS assisted MWA in our team. [file 12885_2020_6519_MOESM1_ESM.doc]

| Table S1 Published literature related to 3DVAPS assisted MWA in our team | |
| --- | --- |
| Authors | Reference |
| Liu F. et al. | A three-dimensional visualisation preoperative treatment planning system in microwave ablation for liver cancer: a preliminary clinical application. nt J Hyperthermia. 2013 Nov;29(7):671-7. doi: 10.3109/02656736.2013.834383. Epub 2013 Sep 20. |
| Liu F. et al. | A three-dimensional visualization preoperative treatment planning system for microwave ablation in liver cancer: a simulated experimental study. Abdom Radiol (NY). 2017 Jun;42(6):1788-1793. doi: 10.1007/s00261-017-1065-z. |
| Li X. et al. | Ultrasound-guided percutaneous microwave ablation assisted by three-dimensional visualization operative treatment planning system and percutaneous transhepatic cholangial drainage with intraductal chilled saline perfusion for larger hepatic hilum hepatocellular (D ≥ 3 cm): preliminary results. Oncotarget. 2017 Jul 15;8(45):79742-79749. |
| Li X. et al. | The value of 3D visualization operative planning system in ultrasound-guided percutaneous microwave ablation for large hepatic hemangiomas: a clinical comparative study. BMC Cancer. 2019 Jun 7;19(1):550. doi: 10.1186/s12885-019-5682-5. |
| Ren H. et al. | Ultrasound-guided percutaneous microwave ablation assisted by a three-dimensional visualization treatment platform combined with transcatheter arterial chemoembolization for a single large hepatocellular carcinoma 5 cm or larger: a preliminary clinical application. Int J Hyperthermia. 2018 Oct 25:1-11. doi: 10.1080/02656736.2018.1530459 |
| Zhang D. et al. | Multiple antenna placement in microwave ablation assisted by a three-dimensional fusion image navigation system for hepatocellular carcinoma. Int J Hyperthermia. 2019 Jan 1;35(1):122-132. doi: 10.1080/02656736.2018.1484183. Epub 2018 Oct 9. |
