# Supplementary figures and images for: 3D visualization ablation planning system assisted microwave ablation for hepatocellular carcinoma (Diameter >3): a precise clinical application
Source: BMC Cancer. 2020 Jan 20;20:44. doi: 10.1186/s12885-020-6519-y (PMC6972027; doi:10.1186/s12885-020-6519-y)

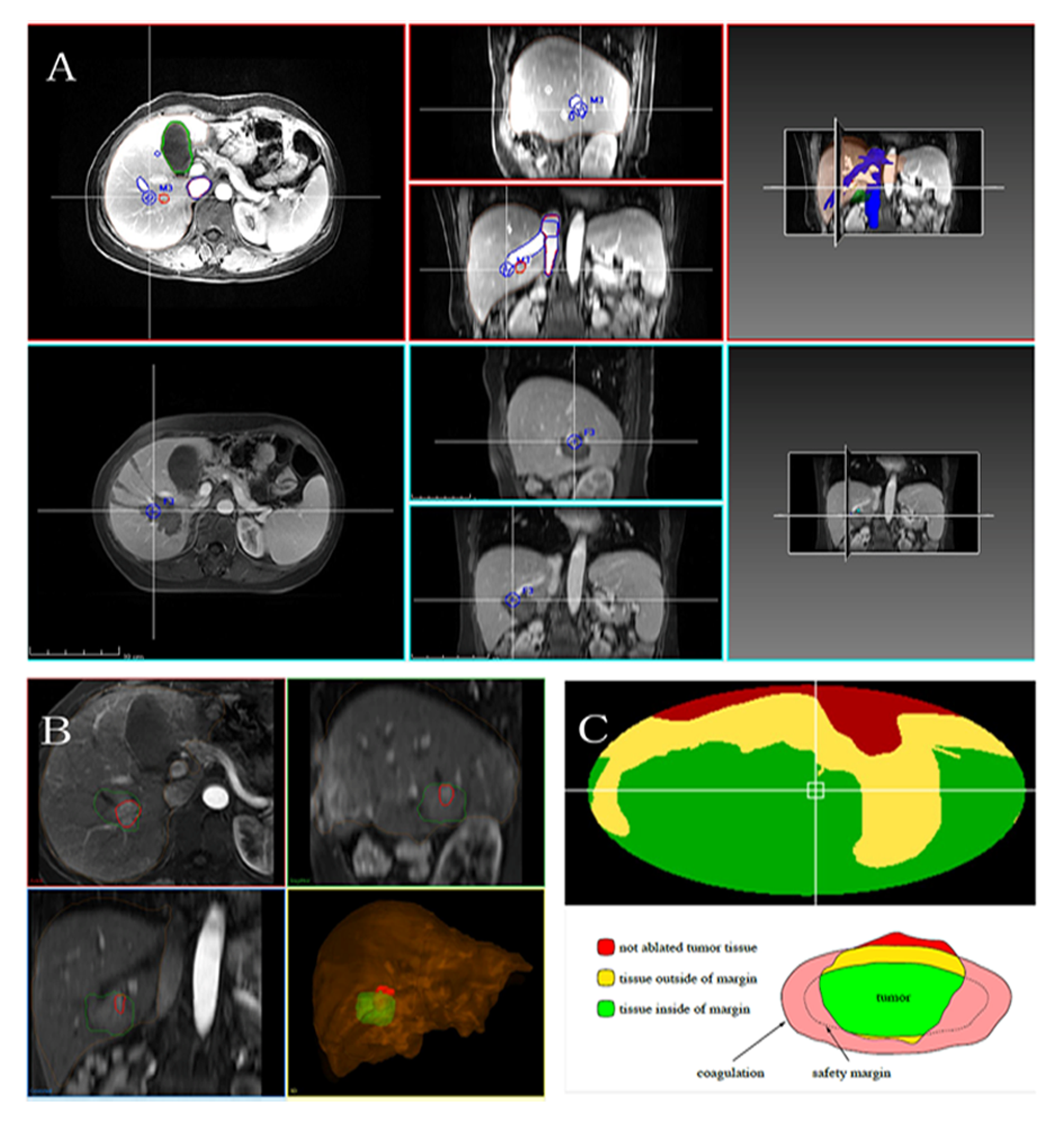

Supplement: Supplementary file 2 — Additional file 2: Figure S1. A picture of the procedure used for 3D visualisation image fusion and tumor map generation. [file 12885_2020_6519_MOESM2_ESM.tif]
